# Efficacy and safety of Tian Meng oral liquid for the treatment of insomnia: A protocol for systematic review and meta-analysis

**DOI:** 10.1097/MD.0000000000026865

**Published:** 2021-08-13

**Authors:** Xinyu Liu, Jiguo Yang, Lu Zhang, Runmin Li, Bingchen Li, Xin Ge, Yuanxiang Liu

**Affiliations:** a College of First Clinical Medicine, Shandong University of Traditional Chinese Medicine, Jinan, People's Republic of China; b College of Traditional Chinese Medicine, Shandong University of Traditional Chinese Medicine, People's Republic of China; c Department of Neurology, Affiliated Hospital of Shandong University of Traditional Chinese Medicine, People's Republic of China; d College of Acupuncture and Massage, Shandong University of Traditional Chinese Medicine, Jinan, People's Republic of China.

**Keywords:** insomnia, meta-analysis, protocol, systematic review, Tian Meng oral liquid, traditional Chinese medicine

## Abstract

**Background:**

The incidence of insomnia is very high and seriously affects the lives of its patients. Long-term insomnia can induce other diseases and even cause sudden death. Tian Meng oral liquid is a type of Chinese patent medicine that has been used in the clinical treatment of insomnia and has certain clinical effects. However, its wide application is limited by the lack of evidence-based medical evaluation.

**Methods:**

Using retrieval strategies, randomized controlled trials on Tian Meng oral liquid for insomnia were obtained from China National Knowledge Infrastructure, WanFang, Chinese Scientific Journals Database PubMed, Embase, and Cochrane Library, regardless of publication date or language. Studies were screened based on inclusion and exclusion criteria, and the Cochrane risk bias assessment tool was used to evaluate the quality of the studies. The meta-analysis was performed using RevMan 5.3 and STATA 14.2 software. Ultimately, the evidentiary grade for the results will be evaluated.

**Results:**

This study aims to evaluate the efficacy and safety of Tian Meng oral liquid in the treatment of insomnia and to provide a more reasonable option for clinical medication.

**Conclusion:**

Our findings will provide a strong basis for the effectiveness and safety of Tian Meng oral liquid in the treatment of insomnia.

## 1 Introduction

According to the 2017 “Guidelines for the Diagnosis and Treatment of Insomnia in Adults in China,”^[1]^ insomnia refers to the subjective experience of feeling dissatisfied with sleep time and/or quality despite having suitable sleep opportunities and sleeping environment, and affects social function. The clinical symptoms include difficulty falling asleep, sleep maintenance disorder, early awakening, decreased sleep quality, and total sleep time reduction, accompanied by daytime dysfunction such as fatigue and physical discomfort. The incidence of this disease is very high.

Epidemiological data show that 45.4% of respondents in China have experienced varying degrees of insomnia in the past month.^[2]^ Long-term insomnia often causes the body's immunity and memory to decline and produces negative emotions such as fear and anxiety. At the same time, insomnia can easily induce high blood pressure, neurasthenia, and even sudden death.

The treatment of insomnia in modern medicine is divided into psychological education, drug therapy, and physical therapy.^[3]^There are some difficulties in the development of psychotherapy.^[4,5]^The main therapeutic drugs include benzodiazepine receptor agonists, melatonin receptor agonists, sedative antidepressants, and orexin receptor antagonists.^[6]^According to the 2017 “Guidelines for the Diagnosis and Treatment of Insomnia in Adults in China,”^[1]^ drug treatment can improve the symptoms of patients, but there are adverse reactions such as dizziness, hypotonia, and fatigue. After stopping the drug, withdrawal symptoms and rebound insomnia will occur. Traditional Chinese medicine (TCM) has made up for these shortcomings to a large extent and has been widely considered in our country.

TCM believes that insomnia belongs to the category of “bu mei,” with rich theoretical experience and reliable curative effects. Tian Meng oral liquid is a type of Chinese patent drug that has the functions of nourishing qi and the kidney, strengthening the spleen and stomach, nourishing the heart, and calming nerves.^[7]^ According to “Tian Meng Oral Liquid (Capsule) Clinical Application Expert Recommendations,”^[8]^it has calming and anti-anxiety effects, promotes gastrointestinal digestion, and can systematically regulate the 3 major functions of the nerve, endocrine, and immune systems. Existing studies have shown that Tian Meng oral liquid can improve patients’ sleep status, reduce their Pittsburgh Sleep Quality Index score, and lead to fewer adverse reactions and high compliance. ^[9,10,11,12]^ Studies have shown that the 18 active ingredients of Tian Meng oral liquid act on 267 target proteins. PTGS2, HSP90AB1, PTGS1, NCOA2, and CALM are the main targets of Tian Meng oral liquid. ^[13]^ Experiments have shown that Tian Meng oral liquid can reduce blood inflammatory factors, increase 5-HT and GABA contents in the raphe nuclei and hippocampus of sleeping rats, and promote the expression of neurotrophic factor in the hippocampus. ^[8,14]^ Therefore, we propose a program to systematically evaluate the efficacy and safety of Tian Meng oral liquid for insomnia.

## 2 Methods

Our protocol has been registered on the International Platform of Registered Systematic Review and Meta-Analysis Protocols. The registration number was INPLASY202170035 (DOI: 10.37766/inplasy2021.7.0035). We strictly abided by Preferred Reporting Items for Systematic Review and Meta-Analysis Protocols guidelines.^[15]^

### 2.1 Data sources and retrieval strategy

Studies were obtained from the China National Knowledge Infrastructure, Wan Fang Data, Chinese Scientific Journals Database, PubMed, Embase, and Cochrane Library, regardless of publication date or language.

The databases were searched by combining the subject words with random words. The retrieval strategy is shown in Table 1 using PubMed retrieval as an example. The search terms were adapted appropriately to conform to different syntax rules of different databases.

**Table 1:** Retrieval strategy of PubMed.

Number	Term
#1	“Sleep Initiation and Maintenance Disorder”[MeSH Terms] OR “Disorders of initiating and maintaining Sleep”[Title/Abstract] OR “Disorders of initiating and maintaining sleep”[Title/Abstract] OR “Early awakening”[Title/Abstract] OR “Nonorganic insomnia”[Title/Abstract] OR “Insomnia”[Title/Abstract] OR “Primary insomnia”[Title/Abstract] OR “Transient insomnia”[Title/Abstract] OR “Rebound insomnia”[Title/Abstract] OR “Secondary insomnia”[Title/Abstract] OR “Sleep initiation dysfunction” [Title/Abstract] OR “Sleeplessness”[Title/Abstract] OR “Insomnia disorder”[Title/Abstract] OR “Chronic insomnia”[Title/Abstract] OR “Psychophysiological insomnia”[Title/Abstract]
#2	“Tian Meng Oral liquid” [Title/Abstract] OR “Sweet Dream Oral Liquid”[Title/Abstract]
#3	Randomized controlled trial [Title/Abstract] OR Controlled clinical trial [Title/Abstract]
#4	#1 AND #2 AND #3

### 2.2 Eligibility criteria

The PICOS principles were given full consideration to establish the inclusion and exclusion criteria of this systematic review.

#### 2.2.1 Type of participants

Regardless of age or sex, patients met the diagnostic criteria for insomnia in the 2017 “Guidelines for the Diagnosis and Treatment of Insomnia in Adults in China.”^[1]^

#### 2.2.2 Type of interventions and comparators

The treatment group was given Tian Meng oral liquid on the basis of conventional treatment, and the control group was given only conventional treatment. Conventional treatment refers to sedation and oral medications such as zopiclone, dexzopiclone, alprazolam, estazolam, and clonazepam.

#### 2.2.3 Type of outcomes

The main results included falling asleep time, sleep duration, sleep quality, physical and mental fatigue after waking up, daytime function, Pittsburgh Sleep Quality Index score, fatigue scale-14 score, and adverse events incidence rate.

#### 2.2.4 Type of studies

The included studies were randomized controlled trials in this systematic review regardless of publication status and language. Animal trials, clinical experience, case reports, and studies with inaccurate designs or incomplete data were excluded.

### 2.3 Study selection and data extraction

**Figure 1:**
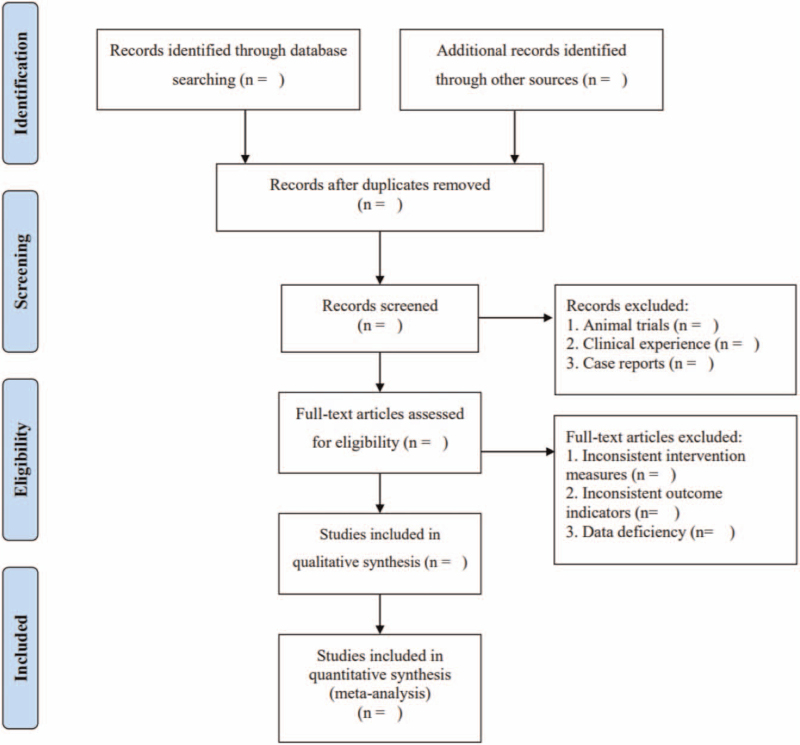
PRISMA flow chart. PRISMA = Preferred Reporting Items for Systematic Review and Meta-Analysis.

EndNote X9 was used to manage the retrieved studies. As shown in Figure 1, the study selection was divided into 2 steps, which were completed by 2 researchers (RL and BL). Preliminary screening involved eliminating repeated and unqualified studies by reading the title and abstract. Rescreening involved reading through the full text and selecting the studies according to the inclusion and exclusion criteria.

According to the Cochrane Handbook for Systematic Reviews of Intervention, the 2 researchers (LZ and XG) extracted the author, publication time, participant number, age, sex, intervention measures, course of disease/treatment, and outcome indicators, filled in the data extraction table, and compared them with each other.

### 2.4 Risk of bias assessment

Two researchers (RL and LZ) assessed the quality of the included randomized controlled trials independently by utilizing the Cochrane risk of the bias assessment tool. As specified by the Cochrane Handbook V.5.1.0, the following sources of bias were considered: random sequence generation, allocation concealment, participant blinding, outcome assessor blinding, incomplete outcome data, selective reporting, and other sources of bias. Each domain was rated as having a high, low, or unclear risk of bias as appropriate. ^[16]^ The 2 reviewers resolved any disagreements through discussion, and a third reviewer (XL) was involved if a consensus could not be reached.

### 2.5 Statistical analysis

The meta-analysis was performed with Review Manager 5.3 and STATA 14.2 software. The outcomes were mainly represented by the mean difference or odds ratio with 95% confidence intervals, and a *P* value <.05 was considered significant. The Cochrane *Q*-test and *I*^2^ statistics were used to assess heterogeneity. When *P* < .1 or *I*^2^ > 50% indicated statistical heterogeneity, a random-effects model was used to calculate the outcomes; otherwise, the fixed-effect model was considered.

### 2.6 Subgroup analysis and publication biases

If there was high heterogeneity in the studies, we performed subgroup analyses to explore the differences in age, sex, interventions, and course of disease/treatment.

We used funnel plots to identify whether there was a small study bias if 10 or more studies were included. The asymmetry of funnel plots suggests the possibility of small-study effects, and the results of the analysis were explained cautiously.

### 2.7 Sensitivity analysis

To ensure the robustness of the combined results, sensitivity analyses were performed to assess the impact of studies with a high risk of bias. We compared the results to determine whether low-quality studies should be excluded.

### 2.8 Quality of evidence

The Grading of Recommendations, Assessment, Development, and Evaluation approach was used to evaluate evidence quality. Considerations of evidence quality assessment include study limitation, consistency of effect, imprecision, indirectness, and publication bias. The evidence quality was classified into 4 levels (high, medium, low, and very low).^[17]^

### 2.9 Ethics and dissemination plans

Given that there were no patients recruited and no data gathered from patients, ethical approval was not necessary for our research. We will publish the results of this meta-analysis in the form of journal papers or conference papers.

## 3 Discussion

TCM has a profound understanding of the disease. As early as 2000 years ago, there was a record of the disease in the “Huang Di Nei Jing,” which was called “mu bu ming” and “bu de wo.” After that, medical scholars from past dynasties contributed various viewpoints and gained much knowledge. Overall, TCM believes that the causes of insomnia are mainly dietary irregularities, emotional disorders, work-rest disorders, and physical weakness after illness. In general, the pathogenesis is excessive Yang leading to Yin deficiency and an imbalance of Yin and Yang. TCM treatment emphasizes “treatment in accordance with 3 categories of etiologic factors,” that is, according to time, place, and person. According to different seasons and climatic factors, different geographical environments, and different physiques, different treatment programs are given, and the curative effect is definite. To reduce the risk of other diseases related to insomnia and alleviate the suffering of patients, the treatment of insomnia should be taken seriously. Tian Meng oral liquid, as adjuvant therapy for TCM, has been used in the clinical treatment of insomnia and has certain clinical effects. The lack of evidence-based medical evaluation limits its wide application. However, similar to potential shortcomings in this systematic review, there may be a heterogeneity risk due to different nationalities, the age of the patient, and a small sample size. Research on the efficacy and safety of Tian Meng oral liquid will provide a more reliable basis for future clinical decision-making and guide development.

## Author contributions

**Conceptualization:** Xinyu Liu, Jiguo Yang, Yuanxiang Liu.

**Data curation:** Lu Zhang, Runmin Li, Bingchen Li, Xin Ge.

**Formal analysis:** Xinyu Liu, Jiguo Yang.

**Methodology:** Yuanxiang Liu.

**Software:** Runmin Li, Bingchen Li.

**Supervision:** Yuanxiang Liu.

**Writing – original draft:** Xinyu Liu and Jiguo Yang.

**Writing – review & editing:** Yuanxiang Liu.
